# Factors associated with HIV counseling and testing behavior among undergraduates of universities and vocational technical training schools in Tbilisi, Georgia

**DOI:** 10.1186/s12889-015-1760-z

**Published:** 2015-04-28

**Authors:** Mamuka Djibuti, Tamar Zurashvili, Tamar Kasrashvili, Carla J Berg

**Affiliations:** RTI International, Georgia HIV Prevention Project, Tbilisi, Georgia; International School of Public Health, Tbilisi State Medical University, Tbilisi, Georgia; Partnership for Research and Action for Health, Tbilisi, Georgia; Department of Behavioral Sciences & Health Education, Emory University Rollins School of Public Health, Atlanta, GA USA

**Keywords:** HIV, AIDS, HIV counseling and testing, Youth, Stigma, Georgia

## Abstract

**Background:**

Stigmatizing attitude towards HIV/AIDS alongside other factors such as HIV knowledge, substance use, sexual behavior, and involvement in various social activities (e.g., internet use, exposure to media) may be related to likelihood of having HIV counseling and testing (HCT). Thus, we examined these associations among 18–24 year old post-secondary school students in Tbilisi, Georgia.

**Methods:**

We conducted a secondary data analysis of a 2010 cross-sectional survey of 1,879 secondary and post-secondary school students aged 15 to 24 years in Tbilisi, Georgia examining sociodemographics, substance use, sexual behavior, HIV-related knowledge and stigmatizing attitudes, and recreational activities in relation to lifetime HCT. A stratified two-stage cluster sample design was used by the parent study with universities selected with probabilities proportional to their size at the first stage, and with a random selection of students stratified by gender in each of the participating university at the second stage.

**Results:**

The vast majority (95.6%) of participants never received HCT. In the multivariate regression model, significant predictors of lifetime receipt of HCT included being married (p = 0.03), not having HIV stigmatized attitude (p = 0.03), more often reading fiction literature (p = 0.02), more often going out in the evenings (p = 0.03), and more often passing time with friends (p = 0.05).

**Conclusions:**

Intervening on HIV stigmatizing attitudes may be a critical prevention or HCT promotion strategy among youth in Georgia. In order to better inform policy and programs, future research should examine contextual factors in secondary and post-secondary schools that impact HCT among Georgian youth. Specifically, factors impacting differential rates of HCT among males and females, the social stigma and knowledge related to HCT and HIV, and the impact of leisure time activity involvement on HCT should be examined further. In addition, interventions and policies that might impact attitudes toward HIV and HCT should be investigated and considered.

## Background

In 2012, 22.0 new HIV diagnoses per 100,000 population were reported in WHO East European Region, and 10.6% of these newly diagnosed cases of HIV infection were in those aged 15–24 years [1]. Georgia (population 4.5 m), one of the former Soviet countries belonging to this region, reported 11.7 newly diagnosed HIV cases per 100,000 population in 2012, and 7.4% of these newly diagnosed cases of HIV infection were in those aged 15–24 years [2]. This calls for appropriate action for the development and implementation of effective prevention interventions of adequate quality and coverage in order to be able to avert new HIV cases among youth [3].

Furthermore, there must be a focus on young people who inject drugs (PWID), young sexual partners of PWID, young sex workers, young men who have sex with men (MSM), young migrants form high prevalence regions, and young people in correctional and prison settings, as these key populations are at higher risk of HIV exposure [4]. Commonly accepted interventions used in the best international practice among youth include prevention education about risks and skills to prevent HIV infection, promotion of condom use with multiple sexual partners, and promotion of HIV counseling and testing (HCT), as the testing is the first step to getting medical care and treatment that can improve health, save lives, and prevent the spread of HIV [5,6].

Global HIV data show that the prevalence of having been tested for HIV among youth significantly varies across different regions, though still remains low. Among youth aged 15–19, highest prevalence of HCT was reported in Eastern and Southern Africa (>20%), followed by West and Central Africa and Latin America and the Caribbean (10 to 20%), and Asia and the Pacific (<10%); very limited data is available from Eastern Europe and Central Asia. HCT prevalence among youth of 15–19 years of age was reported for Kazakhstan (14%) and Armenia (1%) only [7]. Among sexually active high school students in Bosnia and Herzegovina, Former Yugoslav Republic of Macedonia, Serbia, and Montenegro, 5.9% had been tested for HIV [8]. In United States, 12.9% of students were found to be ever tested for HIV in 2013 [9].

In order to improve HCT uptake among youth, it is critically important to know factors affecting HCT that can be addressed through targeted interventions. Limited evidence is available from studies published in international peer review journals – according to the results of two studies from Ethiopia, one study from Ghana, and one study from South Africa conducted among university/college students, individual level factors independently associated with having been tested for HIV include being sexually active, having a boy/girlfriend, never been married, willing to pay for test, risk perception and knowledge about HIV, having ever talked to the mother or female guardian about HIV, and having ever been pregnant or made someone pregnant [10-13]. Among sexually active African American adolescents in four U.S. cities, the strongest predictor of HIV testing was STI knowledge and having talked about testing with partner [14]. There was one study from Ethiopia, which found that perceived stigma associated with the positive test result was significant predictor of low utilization of HCT [10]. Determinants of HCT among youth are largely understudied in Eastern Europe and Central Asia. To the best of our knowledge, there has been just one Balkans study published in international peer reviewed literature, which showed that knowing a friend or relative with HIV, poor self-assessed health status, suspicion of having had an STI, and not having used a condom at first sex were independently associated with HCT [8]. Recent Youth Behavioral Surveillance Survey (BSS) conducted among post-secondary school students 18–24 years of age in the capital of Georgia Tbilisi revealed that only 4.4% of respondents had ever been tested for HIV [15]. We conducted secondary analysis of the data obtained within the framework of this study with the specific aim to explore the role of stigmatizing attitude towards HIV/AIDS alongside factors such as HIV knowledge, substance use, sexual behavior, risky sexual behavior, and involvement in various social activities including having access to internet and printed media in relation to probability of having HCT among 18–24 year old post-secondary school students in Tbilisi, Georgia.

## Methods

### Participants and procedures

The current study is a secondary data analysis of the 2010 USAID-funded Georgia HIV Prevention Project Behavioral Surveillance Survey among School and University Students in Tbilisi [15]. The Georgia HIV Prevention Project began February 4, 2010 and ended August 31 2014, which was designed to improve and expand upon HIV prevention among key affected populations. This study was conducted among 15–18 year old secondary school students and 18–24 year old post-secondary school students in Tbilisi in order to fill the gap in current data about the knowledge, attitudes, and behaviors of youth in the location of the highest number of reported HIV/AIDS cases – Tbilisi, the capital of Georgia. However, for the current analysis we used the data for only 18–24 year olds participants, as in-depth data on risky behaviors including sexual practice and drug use were collected among this age group only. Thus, the statistical population of the parent study was all students 18–24 years of age who were: a) undergraduates in 36 private and 10 public universities; and b) students in 7 vocational-technical training schools (VTTSs) in Tbilisi in 2011 (per the Ministry of Education and Science as of September 2010). The total number of post-secondary students was 73,652 in 46 universities and 7 VTTSs in Tbilisi.

A stratified two-stage cluster sample design was used. The sample size calculation was done using the methodology for descriptive studies for an expected response proportion of 50% (Confidence Interval [CI] of 0.10; Confidence Level of 95%), indicating a minimum of 384 students in each of the two gender groups. Next, estimation of the sample size was done for a comparison of proportions of dichotomous variables for alpha error = 0.05 (two-sided test), power = 80%, and the expected smaller proportion = 0.5 (again maximizing the sample size) for the detection of difference = 0.10. By this methodology it was determined that a minimum of 407 students should be selected to reach an adequate statistical significance for mutual comparisons of age and gender groups. Therefore, this study attempted to enroll 1,000 students in total, 500 per gender group, considering a potential 20% rate of non-response.

For the first sampling stage, universities/VTTSs were sampled with probabilities proportional to their size (PPS) from the list of all 46 universities and 7 VTTSs located in Tbilisi (the planned number of study subjects – 1000; 50 students per primary sampling unit; 20 primary sampling units; the sampling interval 3,683 [i.e. =73,652/20], the random starting point selected by random number generator 1557). As a result, 13 universities and/or VTTSs (7 state universities, 5 private universities, and one VTTS) were selected at the first stage. In very large universities, more than one unit was selected. The second stage of sampling involved a random selection of students (not classes) stratified by gender in each of the participating university/VTTS. The lists of eligible students were created. Restriction criteria were defined by age (18–24 years) and knowledge of Georgian. The simple random sampling technique was applied in selection of the study subjects from the lists of the eligible students. In each unit, 25 female and 25 male subjects were selected from the corresponding lists (two lists generated by gender).

The BSS protocol was reviewed and approved by two Institutional Review Boards (IRBs), RTI’s IRB and the local IRB of the Maternal and Child Care Union in Georgia, to ensure the rights and welfare of participants were protected. All pupils and students were informed of the nature of the study prior to their participation. For school pupils (15–17 years) both written parental consent and the pupil’s written consent were obtained. For students (18–24 years) written consent was obtained. In addition, the youth were informed that at any time during the interview they had the freedom to refuse to answer a question or to discontinue the interview. All respondents were informed that their participation was voluntary and that their responses would remain anonymous.

Refusal rate for post-secondary students was 1.3%. The actual sample achieved was 987. Among these 987 completed questionnaires, 25 were further excluded because of ineligible ages (i.e., more than 24 years of age) of respondents or because the questionnaires were incomplete (i.e., almost half of the questions were left unanswered), leaving a total of 962 useable questionnaires.

### Conceptual framework

For guiding the analysis, we used the Socio Ecologic Model (SEM), which is a framework to examine the multiple effects and interrelatedness of environmental, contextual, and social factors on individual behavior. Recognizing that most public health challenges are too complex to be adequately understood and addressed from single level analyses, the SEM includes a more comprehensive approach that integrates multiple levels of influence to impact health behavior and ultimately health outcomes. Those levels of influence include intra- and interpersonal factors, community and organizational factors, and public policies [16-18]. For example, some variables that might influence HCT may be intrapersonal factors such as socio-demographics, substance use behaviors, HIV knowledge, and stigmatizing attitudes toward HIV/AIDS; interpersonal factors such as social exposure to HIV positive persons or high risk groups; community or organizational factors such as prevalence of HIV in their community or social norms within their community manifested as stigma and discrimination against people living with HIV; or public health policies including those that regulate confidential and anonymous HCT. Drawing from this perspective in the context of using an existing data set (i.e., no assessment of community/organizational factors), the current study aims to examine individual factors (e.g., socio-demographics, substance use behaviors, HIV knowledge, and stigmatizing attitudes toward HIV/AIDS) and interpersonal factors (e.g., involvement in activities with the potential for social influence on HCT) among youth in Tbilisi, Georgia.

### Measures

The survey assessed the aforementioned factors drawing from the SEM. Specifically, the following assessments were included:

- *HIV Counseling and Testing (HCT)*. The primary outcome of interest for our analyses was the probability of having HCT. Participants were asked, “We are not asking about the result, but have you ever had HIV test?” This variable was measured as a dichotomous variable with a value of one indicating that the respondent has had an HIV test and zero indicating that they never had an HIV test that included those who answered no and assuming the worst scenario those who answered don’t remember and refused to answer.
- *Socio-demographics*. Participants were asked to indicate their age, gender, employment status, and marital status.
- *Cigarette Smoking*. Participants were asked, “Have you ever smoked a cigarette?” and “How often were you smoking cigarettes over the last month? Have not smoked at all; less than 1 cigarette per week; less than 1 cigarette per day; 1–5 cigarettes per day; 6–10 cigarettes per day; 11–20 cigarettes per day; or more than a pack a day.” These measures were adapted from the Global Youth Tobacco Survey [19].
- *Alcohol Use*. Participants were asked, “Have you ever had an alcoholic drink (wine, beer, vodka, martini, champaign, other drink containing alcohol)?” and “Have you had an alcoholic drink over the past month?” These questions are adapted from other validated surveys [20,21]. For the current analysis, we used the latter question to indicate alcohol use given the high prevalence of lifetime use (91.9%) versus past 30-day use of alcohol (64.8%).
- *Marijuana Use*. Participants were asked, “On how many occasions (if any) have you smoked marijuana or hashish - In your lifetime? In the past 12 months? In the past 30 days?” These assessments were adapted from other validated surveys. Given the low prevalence of past 30 day use (1.0%) and past 12 month uses (4.3%), lifetime marijuana use (11.1%) is included in the current analyses.
- *Ecstasy Use.* Participants were asked, “On how many occasions (if any) have you used ecstasy?” In your lifetime? In the past 12 months? In the past 30 days?” These assessments were adapted from other validated surveys. Given the low prevalence of past 30 day use (1.1%) and past 12 month uses (2.0%), lifetime ecstasy use (3.4%) is included in the current analyses.
- *Sexual Behavior.* Participants were asked, “Have you ever had sexual intercourse?” with response options of “*yes”* and *“no”.*
- *Risky Sexual Behavior.* This variable was computed from four different variables: (1) participants were asked “Did you use condom with sex worker during last 12 month? With response options “yes”, “no”, “don’t remember” and “refused to answer”. Those who answered no and assuming the worst scenario those who answered don’t remember and refused to answer were defined as having risky behavior; (2) “Did you use condom with occasional partner during last 12 month? with response options “yes”, “no”, “don’t remember” and “refused to answer” Those who answered no and assuming the worst scenario those who answered don’t remember and refused to answer were defined as displaying risky behavior; (3) How many sex partners did you have in the last 12 months? With response options “one”, “two”, “three”, “up to 5”, “5 to 10”, “more than 10”, “don’t remember” and “refused to answer” and (4) how often did you use condom during last 12 months? with response options “always”, “almost always”, “rarely”, “never”, “don’t know” and “refused to answer” – answers two and more partners including don’t remember and refused to answer for number of sex partners in combination with almost always, rarely, never, don’t know and refused to answer for condom use were defined as engaging in risky behavior.
- *HIV Knowledge*. Participants were asked to give their opinion about each of the following 11 questions: “Can the risk of HIV transmission be reduced by having sex with only one uninfected partner who has no other partners? Can a person reduce the risk of getting HIV infection by using a condom every time he or she has sex? Can a healthy looking person have HIV? Can a person get HIV from a mosquito bite? Can a person get HIV infection through kissing? Is it possible for a person to get HIV in a beauty salon, by using tools used by other people during manicure, pedicure, or tattoo? Can a person get HIV by sharing food with someone who is infected? Can a person get HIV if s/he uses the needle/syringe that was previously used by another person? Can a fetus get HIV from an HIV-infected mother during pregnancy? Can a fetus get HIV infection from an HIV-infected mother during delivery? And can a baby get HIV from an HIV-infected mother through breast-feeding?” Response options included “yes”, “no”, and “do not know”, based on which the HIV knowledge score was calculated (for each question, a correct answer was given 1 point,, uncertainty (“do not know”) and a wrong answer – 0 point).
- *HIV Stigmatizing Attitude.* Participants were asked, “If a relative of yours became infected with HIV, would you want it to be kept a secret?” with response options of “yes”, “no”, and “do not know” based on which the stigmatizing attitude was defined if participant responded “yes” or “do not know”, and not stigmatizing attitude was defined if participant responded “no”. This question was selected in the original survey instrument measuring stigmatizing attitude on the key conceptual domain of social judgment including shame, blame, prejudice and stereotypes [22,23].
- *Activity Involvement*. Participants were asked about the frequency with which they engaged in several activities, which ranged in the degree to which social influence on HIV testing may be involved. These six items asked, “How often have you done the following: Read fiction literature for entertainment? Engaged in sports or physical exercising? Went to parties, cafes, bars, or disco in the evening? Used the internet to listen to music, play, or chat? Used the internet for educational or work purposes? And went out in the neighborhood street and pass time with neighborhood friends or neighbors?” Response options ranged from 1 = never to 5 = almost every day*.* These measures were adapted from the The European School Survey Project on Alcohol and Other Drugs [24].

### Statistical analysis

Participant characteristics were summarized using descriptive statistics. Bivariate analyses were conducted comparing those who ever had HCT with those who never had HCT among 18–24 year old post-secondary school students. Chi-squared tests were used for categorical variables, and independent samples t-tests were used for continuous variables. Binary logistic regression was used to examine factors associated with the likelihood of receiving HCT during lifetime among 18–24 year old post-secondary school students. We forced age and all factors that were associated with HCT at the p < .10 in bivariates for the regression. SPSS 21.0 was used for all data analyses. Statistical significance was set at α = .05 for all tests.

## Results

For students (18–24 years), the refusal rate was 1.3% (13 of the 1000 solicited students refused). The initial sample achieved was 987. Among these 987 completed questionnaires, 25 were excluded because of ineligible age (more than 24 years of age) or because they were incomplete (almost half of the questions were left unanswered), leaving a total of 962 useable questionnaires. Though the sample was Tbilisi students 18–24 years of age, almost one-half of the student respondents were from the various regions in Georgia, allowing more generalizability of the study results.

Table 1 summarizes participant characteristics and bivariate analyses examining differences between participants who received HCT versus who had not during their lifetime. In terms of receiving HCT during lifetime, the vast majority (95.6%) of participants never received HCT. Among all participants, 91.9% showed stigmatizing attitude; this tendency was significantly more likely for women than for men (94.4% vs. 89.0%; p = 0.00; not shown in tables).

**Table 1:** **Participant characteristics among 18–24 year olds enrolled in a post-secondary school in Tbilisi and bivariate analyses examining those who received HCT during their lifetime versus not, N = 962, (based on the data collected through the parent study in 2010)**

**Variable**		**Received HCT**		
	**Total**	**No**	**Yes**	
	**N=962**	**N=857**	**N=39**	
	**100%**	**95.6%**	**4.4%**	
	**Mean (SD)**	**Mean (SD)**	**Mean (SD)**	**p**
	**or N (%)**	**or N (%)**	**or N (%)**	
*Sociodemographics*				
Age (SD)	20.4 (1.3)	20.41 (1.2)	20.5 (1.6)	0.69
Gender (%)				**0.00**
Male	462 (48.0)	383 (93.0)	29 (7.0)	
Female	500 (52.0)	474 (97.9)	10 (2.1)	
Employment (%)				0.26
Unemployed	797 (82.8)	718 (96.0)	30 (4.0)	
Employed at least part-time	165 (17.2)	139 (93.9)	9 (6.1)	
Marital status (%)				0.06
Not married	924 (96.0)	826 (95.9)	35 (4.1)	
Married	38 (4.0)	31 (88.6)	4 (11.4)	
*Substance Use*				
Cigarette use, past month (%)				0.43
No	210 (43.8)	191 (95.0)	10 (5.0)	
Yes	270 (56.2)	235 (93.3)	17 (6.7)	
Alcohol use, past month (%)				**0.00**
No	268 (30.2)	251 (98.4)	4 (1.6)	
Yes	619 (69.8)	547 (94.3)	33 (5.7)	
Marijuana use, lifetime (%)				0.37
No	15 (8.2)	12 (85.7)	2 (14.3)	
Yes	167 (91.8)	140 (91.5)	13 (8.5)	
Ecstasy use, lifetime (%)				**0.02**
No	851 (91.4)	770 (96.5)	28 (3.5)	
Yes	80 (8.6)	63 (90.0)	7 (10.0)	
*Psychosocial Factors*				
Sexually active, lifetime (%)				**0.00**
No	482 (50.2)	459 (98.3)	8 (1.7)	
Yes	478 (49.8)	396 (92.7)	31 (7.3)	
Risky sexual behavior, past year (%)				0.46
No	91 (22.9)	82 (94.3)	5 (5.7)	
Yes	306 (77.1)	247 (91.8)	22 (8.2)	
HIV knowledge (SD)	6.9 (2.6)	6.9 (2.6)	7.0 (2.3)	0.67
HIV stigmatizing attitude (%)				**0.03**
No stigmatizing attitude	71 (8.1)	64 (90.1)	7 (9.9)	
Stigmatizing attitude	807 (91.9)	771 (96.1)	31 (3.9)	
Activity involvement (SD)				
Read fiction literature for entertainment	2.9 (1.2)	2.9 (1.2)	3.4 (1.2)	**0.02**
Engage in sports, physical activity	3.0 (1.4)	3.0 (1.4)	3.2 (1.4)	0.28
Go to parties, café, bar or disco in the evening	2.7 (1.1)	2.7 (1.1)	3.1 (1.1)	**0.00**
Use the internet to listen to music, play, chat	4.3 (1.2)	4.4 (1.1)	4.4 (1.2)	0.98
Use the internet for educational or work purposes	4.1 (1.2)	4.2 (1.1)	4.4 (0.9)	0.11
Pass time w/ neighborhood friends/neighbors	3.0 (1.4)	3.0 (1.4)	3.6 (1.3)	**0.00**

In bivariate analyses (Table 1), males were significantly more likely to have had an HIV test than females (7% vs. 2.1%; p = 0.00). Other correlates of HCT included past 30-day alcohol use (p = 0.00), lifetime ecstasy use (p = 0.02), ever having sexual intercourse (p = 0.00), HIV stigmatizing attitude (p = 0.03), more often reading fiction literature (p = 0.02), more often going out in the evenings (p = 0.00), and more often passing time with friends (p = 0.01).

In the multivariate regression model (Table 2), significant predictors of lifetime receipt of HCT included being married (p = 0.03), not having HIV stigmatized attitude (p = 0.03), more often reading fiction literature (p = 0.02), and more often going out in the evenings (p = 0.03), and more often passing time with friends (p = 0.05).

**Table 2:** **Multivariate model examining predictors of lifetime receipt of HCT among participants aged 18–24 years enrolled in a post-secondary school in Tbilisi (based on the data collected through the parent study in 2010)**

**Variable**	**OR**	**CI**	**p-value**
Age	1.15	0.86-1.54	0.53
Gender			0.07
Male	Ref		
Female	0.28	——0.07-1.13	
Marital status			**0.03**
Not marries	Ref		
Married	4.99	——1.23-20.24	
Alcohol use, past month			0.11
No	Ref		
Yes	2.81	——0.79-9.98	
Ecstasy use, lifetime			0.70
No	Ref		
Yes	1.24	——0.42-3.70	
Sexually active, lifetime		-	0.84
No	Ref		
Yes	0.84	—0.19-3.67	
HIV stigmatizing attitude			**0.03**
Yes	Ref		
No	0.33	——0.12-0.89	
Activity involvement:			
Read fiction literature for entertainment	1.46	1.10-1.99	**0.02**
Go to parties, café, bar or disco in the evening	1.54	1.05-2.24	**0.03**
Pass time with neighborhood friends/neighbors	1.35	1.01-1.82	**0.05**

Abbreviations: OR-odds ratio; CI-confidence interval.

## Discussion

The current study documented sociodemographic factors, substance use, and psychosocial factors, particularly HIV stigmatizing attitudes and knowledge, in relation to HCT among post-secondary students in Tbilisi, Georgia. Key results indicated that being married, not having HIV stigmatized attitude on social judgment domain, more often reading fiction literature, more often going out in the evenings, and more often passing time with friends were significant predictors of HCT. This information is critical, as promotion of HCT is the first step to getting medical care and treatment that can improve health, save lives, and prevent the spread of HIV [5].

Interestingly, being married was a predictor of having had an HIV test. In Georgia, there is no formal policy requiring HIV test before marriage, however to be tested before getting married could be a common practice particularly among young males who mostly undertake a risky sexual behavior [15], who might be willing to get tested before marriage. Further research is required to explain this phenomenon.

Prior research from sub-Saharan Africa and USA suggests that being sexually active, having a boy/girlfriend, never been married, willing to pay for test, risk perception and knowledge about HIV/STI, having talked about testing with partner or the mother/female guardian, and having ever been pregnant or made someone pregnant are important factors associated with increased likelihood of HCT [10-14]. There has been one study from Ethiopia, which found that perceived stigma associated with the positive test result was significantly associated with low utilization of HCT among university students [10]. Importantly, not having an HIV stigmatizing attitude on social judgment domain was associated with HCT in this sample. The current findings are particularly important given that sexual behavior and knowledge regarding HIV were not significant predictors of HCT in the multivariate analyses. Thus, intervening on HIV stigmatizing attitudes on social judgment domain may be a critical prevention or HCT promotion strategy, which may have important policy implications for Georgia and possibly for other in Eastern European and Central Asian countries characterized by similar socio-economic context and HIV epidemiological profile. Namely, in addition to a considerable effort of national governments and international donors to improve HIV knowledge through life skills education among youth, adequate investment should be made to advance fight against HIV associated stigma too.

Finally, leisure time activities including more often reading fiction literature, more often going out in the evenings, and more often passing time with friends were significant predictors of HCT. The reasons for these findings are unclear. Perhaps there is more opportunity for social influence, even vicariously, can influence one’s perceptions regarding HCT and ultimately their openness to receiving it. Another possibility is that those individuals more frequently going out or spending time with peers had more sexual activity. However, having risky sexual behavior during past 12 months did not prove to be an independent predictor for undertaking HIV test which may suggest that alternately, leisure time activities may provide good opportunity to peers to exchange information where to obtain a test and the facts of HIV/AIDS.

The current findings have important implications for research and practice. In terms of research, this study suggests the need for more research regarding correlates of HCT among young adults in Georgia, given the relatively limited scope of factors included in this data set. Specifically, examining the contextual factors in post-secondary schools and the differences among males and females that might contribute to HCT is critical. Moreover, the impact of leisure time activities on attitudes about HIV and HCT and likelihood of HCT should be examined in these contexts and how different groups are targeted. Regarding practice, practitioners should understand the relationship between other behaviors and HCT and systematically monitor health behaviors in clinical encounters. Additionally, addressing these behaviors concurrently may prove to be beneficial.

This study has some limitations. First, the parent study recruited this sample through secondary and post-secondary school students living in Tbilisi, and thus, we cannot infer how reflective this sample is of the larger youth population in Georgia. Relatedly, we also did not record the school of attendance as a variable. This was done in order to ensure maximum confidentiality of the data given the sensitive nature of the questions (e.g., history of drug use, sexual behavior) and to provide the maximum protection of the schools who participated in the study. It is important to note the large number of school involved (i.e., 13 universities and/or vocational-technical training schools) and thus the greater generalizability of the sample to the larger Tbilisi 18–24 year old population and potentially the larger population within this age range in Georgia. Despite these strategies, we are uncertain of the extent to which the lifetime HCT prevalence accurately reflects actual national or city-wide estimates among this population. Second, because of the cross-sectional nature of this study, we cannot determine the directionality of the relationships documented. Furthermore, this was a secondary data analysis of a study examining factors relevant to HIV Prevention; it may not have exhaustively assessed all factors potentially related to HCT. In addition, potential social desirability bias towards risky sexual behaviors might affect the validity of the study findings. Finally, the way in which we operationalized the alcohol and marijuana variables has limitations. However, we examined alternative operationalizations (e.g., continuous variables), and these alternatives did not yield significantly different findings. Despite these limitations, these findings are novel and important as a basis for future research in this area, particularly given the dearth of published research on youth HCT in Georgia and the region.

## Conclusions

Intervening on HIV stigmatizing attitudes may be a critical prevention or HCT promotion strategy, which may have important policy implications for Georgia. Future research should examine contextual factors in secondary and post-secondary schools that impact HCT among Georgian youth. Specifically, factors impacting differential rates of HCT among males and females, the social stigma and knowledge related to HCT and HIV, and the impact of leisure time activity involvement on HCT should be examined further. In addition, interventions and policies that might impact attitudes toward HIV and HCT should be investigated and considered.

## Abbreviations

AIDS: Acquired immunodeficiency syndrome
CI: Confidence interval
HCT: HIV counseling and testing
HIV: Human immunodeficiency virus
IRB: Institutional review board
OR: Odds ratio
SD: Standard deviation
USAID: United States agency for international development
VTTS: Vocational-technical training school
WHO: World Health Organization
